# Intra-household gender difference in dietary diversity and its determinants in a food-insecure community in the Democratic Republic of Congo

**DOI:** 10.11604/pamj.2026.53.129.49323

**Published:** 2026-03-16

**Authors:** Nelly Kabena-Ngandu, Rosa Munsambe-Yambu, Josué Lukusa, Landry Egbende, Alexandre Bukasa-Kanyama, Bernard-Kennedy Nkongolo, Alain Cimuanga-Mukanya, Marie-Claire Muel Telo Muyer, Freddy Bangelesa

**Affiliations:** 1 Department of Nutrition, School of Public Health, Kinshasa University, Kinshasa, Democratic Republic of Congo,; 2 Department of Management, School of Public Health, Kinshasa University, Kinshasa, Democratic Republic of Congo,; 3 Faculty of Medicine, Public Health and Pharmacy, University of Mbujimayi, Mbujimayi, Democratic Republic of Congo

**Keywords:** Food insecurity, dietary diversity, associated factors, households, Democratic Republic of the Congo

## Abstract

**Introduction:**

food insecurity in the Democratic Republic of Congo affects over 25 million people, with Kasaï Oriental Province among the most impacted-approximately 74.9% of households face acute food shortages. While food access has been widely studied, evidence on intra-household, gender-based differences in dietary diversity is limited, particularly in highly food-insecure urban settings. This study aimed to investigate the differences in dietary diversity between men and women in Mbuji-Mayi and to identify the household- and individual-level factors associated with these differences.

**Methods:**

a cross-sectional study was conducted among 360 households selected by multistage random sampling, from Bonzola Health Zone, Mbuji-Mayi City. Data were collected through interviews using an electronic questionnaire. Dietary diversity was assessed using 24-hour dietary recall scores for men and women. Differences in dietary diversity scores were tested using the Mann-Whitney U test. Poisson regression models were used to identify predictors of dietary diversity for men and women, while a generalized linear model was used to assess the determinants of dietary diversity differences within households.

**Results:**

women had significantly higher dietary diversity scores than men (p=0.030). Key factors associated with the difference in dietary diversity scores between women and men (DDS-WM) included wealth index (β=-0.159; p=0.001), household size (β=-0.139; p=0.004), number of meals per day (β=-0.127; p=0.008), education level of the household head (β=-0.146; p=0.003), and the reduced Coping Strategies Index (rCSI) (β=0.100; p=0.029).

**Conclusion:**

these findings revealed disparities in dietary diversity between men and women in Bonzola, and some associated factors were also identified. They highlight the need for gender-sensitive approaches in nutrition policies and programs to ensure equitable dietary outcomes in food-insecure settings.

## Introduction

Over the past decades, global interest has increasingly turned toward the complex challenge of food insecurity - not only as a matter of availability but also as one of equitable access and intra-household distribution [1,2]. Food security, as defined by the Food and Agriculture Organization (FAO), exists when all people, at all times, have physical and economic access to sufficient, safe, and nutritious food that meets their dietary needs and food preferences for an active and healthy life [3]. Yet, according to the latest estimates, around 733 million people faced hunger in 2023, equivalent to one in eleven people globally, and one in five in Africa [4].

Africa remains one of the continents most severely affected, with nearly one in five people experiencing undernourishment [4]. In African urban settings, slum residency, large household size, poverty, low education levels, and older household heads continue to exacerbate food insecurity [5]. The Democratic Republic of Congo (DRC) is among the hardest-hit countries. According to the 2023 Integrated Food Security Phase Classification (IPC), more than 25 million Congolese were in Crisis (IPC phase 3) or worse levels of acute food insecurity, making the DRC one of the most affected nations [6]. In Kasai Oriental, where 74.9% of households, or over 4 million people, were found to be food insecure in 2020, 45.9% of households in the Bonzola Health Zone alone experienced moderate or severe food insecurity [7].

While these statistics underscore the severity of the food insecurity crisis, they often mask the inequalities that exist within households. Most food security assessments assume equal food distribution among household members, as they aggregate data at the household level [1,8]. However, growing evidence challenges this assumption, demonstrating that intra-household disparities, especially those based on gender, can significantly affect individual nutritional outcomes [9-13]. Due to a complex interplay of sociocultural norms, power dynamics, and economic dependency, women and girls frequently have limited access to a diverse range of nutrient-dense foods, particularly in low-income or patriarchal settings [5,10].

These gendered dynamics have been widely documented. For example, in Bangladesh, gender-based food disparities were observed, influenced by socio-economic factors, cultural norms, and local dietary practices [14]. In Ethiopia, intra-household food allocation was shaped by income inequalities, bargaining power, dietary habits, social status, and interpersonal relationships [10].

Yet, in the DRC, and particularly in the Kasai region, research on gender-based nutritional disparities remains scarce, despite clear vulnerabilities among women of reproductive age. In this context, the present study aims to investigate the differences in dietary diversity between men and women in Bonzola Health Zone and to identify the household and individual-level factors associated with these differences.

## Methods

**Study design:** this was a cross-sectional study conducted between May and June 2024. The design incorporated multistage cluster sampling, with households as the unit of analysis.

**Setting:** the study was conducted in the Bonzola Health Zone, located in the commune of Kanshi, in the southern part of Mbuji-Mayi City, Kasaï Oriental Province, Democratic Republic of Congo. The health zone, randomly selected among the urban health zones of the province, consists of 16 health areas and had an estimated population of 236,348 in 2024 [15]. The local economy is primarily driven by artisanal diamond mining, small-scale trade, and past employment in the Bakwanga Mining Company (MIBA). Data collection took place over a two-month period (May-June 2024) through household visits.

**Study population and sampling strategy:** the study population included households residing in the Bonzola Health Zone. Inclusion criteria were households that had lived in the zone for at least six months and had at least two members of different sexes. Households were excluded if the primary respondent was unable to participate due to severe illness or mental impairment.

The minimum sample size of surveyed households was calculated using the following formula [16]:


$$n\geq \frac{2\sigma^{2}{\left(z_{\alpha /2}+z_{1-\beta }\right)}^{2}}{\delta^{2}}$$

Where n is the minimum required sample size; σ is the estimated standard deviation of the diversity score; Z_(α/2)_ is the critical value for the chosen significance level; Z_1-β_ is the critical value associated with the desired statistical power; δ is the expected effect size, defined as the minimum difference in mean dietary diversity scores between men and women to be detected.

Based on previous studies [17-19], the sample size was calculated to detect a minimum mean difference of 0.30 in dietary diversity scores between the two groups, assuming an estimated standard deviation of 0.708, a statistical power of 80% (1 - β = 0.80), and a 95% confidence level (α = 0.05). Under these assumptions, the minimum required sample size was estimated at 175 households for both groups combined. To account for the study design, a design effect of 1.5 was applied, reflecting potential clustering and acknowledging logistical constraints associated with data collection. This adjustment increased the required sample size to 263 households. Furthermore, a correction factor of 1.24 was incorporated to adjust for the inclusion of multiple covariates in the regression model (11 explanatory variables), thereby compensating for the anticipated loss of statistical power. Finally, an additional 10% was added to account for potential non-response and missing data. After applying these adjustments, the final sample size retained for the study was 360 households.

A three-stage random sampling procedure was used. First, three of the 16 health areas were selected using simple random sampling. Second, within each selected health area, six avenues were randomly selected. Third, from each avenue, 20 households were chosen through systematic random sampling. A sampling interval (k) was determined by dividing the total number of households on each avenue by 20. A random starting number between 1 and k was selected to determine the first household, and every k-th household thereafter was included in the sample.

**Data collection:** data were collected using a structured digital questionnaire administered during in-person interviews. Household-level and individual-level information were obtained from the individual primarily responsible for food preparation or distribution. The questionnaire was developed based on established food security and nutrition assessment tools and adapted to the local context. It was implemented using the SurveyCTO Collect mobile application and included closed and semi-open questions. The instrument was pre-tested in a non-selected health area with similar sociodemographic characteristics, and necessary revisions were made prior to final deployment. The development and structure of the questionnaire were informed by previously validated tools [20,21]. Dietary diversity was assessed using a standardized 24-hour recall method following the FAO guidelines [22]. The household wealth index was constructed using principal component analysis (PCA) based on household asset ownership, consistent with established methodologies, and categorized into quintiles [23,24]. The reduced Coping Strategies Index (rCSI) was calculated based on the frequency of five food-related coping behaviors reported over the previous seven days, following the standard methodology developed by the World Food Program [25]. The Household Hunger Scale (HHS) was derived from reports of severe food insecurity experiences over the preceding 30 days and scored according to the Food and Nutrition Technical Assistance (FANTA) guidelines [26].

Enumerators received three days of training covering study objectives, interview techniques, ethical considerations, use of electronic data collection tools, and standardization of dietary recall procedures. Field supervision was conducted daily to ensure adherence to protocols. The digital questionnaire incorporated built-in validation checks, skip patterns, and range restrictions to minimize entry errors. Regular data quality reviews were performed during collection to ensure completeness and consistency, thereby strengthening internal validity. The standardized sampling procedures and uniform implementation across all selected households support the external validity of the findings.

**Study variables and measurements:** the main outcome variables included: (1) Men´s Dietary Diversity Score (MDDS); (2) Women´s Dietary Diversity Score (WDDS); and (3) the intra-household dietary diversity gap, measured as the Difference in Dietary Diversity Scores between Women and Men (DDS-WM). This last indicator has been used in previous studies to evaluate food allocation equity within households [10]. DDS-WM is defined as the difference between women´s and men´s dietary diversity scores within the same household. This metric was used to capture the direction and magnitude of gender-based disparities in dietary diversity at the household level. A positive DDS-WM value indicates higher dietary diversity among women compared to men, whereas a negative value reflects higher dietary diversity among men. A value close to zero suggests relative parity in dietary diversity between sexes. Conceptually, DDS-WM provides a simple and interpretable measure of intra-household dietary distribution, allowing identification of potential gender imbalances in food access and consumption. Therefore, each dietary diversity score was adapted from the FAO Minimum Dietary Diversity for Women (MDD-W) guidelines [22]. It was calculated using a 24-hour food recall covering ten aggregated food groups (cereals, pulses, nuts and seeds, milk and dairy products, meat and fish, eggs, dark green leafy vegetables, other vegetables and fruits rich in vitamin A, other vegetables, other fruits). Consumption of at least one item from a given food group contributed one point to the score (range: 0-10), with a score ≥5 indicating adequate dietary diversity.

Independent variables included: demographic characteristics (gender, age, education level, occupation of the household head), household size, tenure status, daily meal frequency, meal scheduling, wealth index, Coping Strategies Index (CSI), and Household Hunger Index. The gender of the head of household was recorded based on self-identification. Age was calculated in completed years using the date of birth and the date of the interview. Educational attainment was classified according to the highest level of schooling completed and grouped into three categories: primary, secondary, and higher/university education. The head of household´s occupation was defined as their main income-generating activity. Land tenure status was determined by identifying whether the household owned the plot on which their dwelling was built, rented or sublet it, or occupied it through family ownership. Household size was determined by counting all individuals considered members of the household. A household member was defined as any person who recognized the authority of the head of household and regularly shared both residence and meals with the group. Visitors and individuals staying temporarily without the intention of remaining in the household were excluded.

The household wealth index was constructed based on ownership of selected assets. First, weights were assigned to each asset using Principal Component Analysis (PCA). The resulting values were then standardized using the mean and standard deviation. Each household received a standardized score for every asset, depending on whether it was owned. These standardized scores were summed to generate the overall wealth index for each household. Households were subsequently divided into wealth quintiles. Wealth index scores were first ordered in ascending order, after which households were grouped into five equal categories. Absolute and relative frequencies were calculated for each quintile.

The Coping Strategies Index (CSI) was calculated using a simplified seven-day recall period. Households were asked how many days in the previous week they had relied on five common food-related coping strategies, with standardized weights applied [27]. These strategies included: (1) consuming less preferred or less expensive foods; (2) borrowing food or seeking assistance from friends or relatives; (3) reducing portion sizes; (4) reducing adult food intake to prioritize children; and (5) reducing the number of meals consumed per day.

The Household Hunger Scale (HHS) was calculated using a 30-day recall period. Households reported how often they had experienced three severe food deprivation situations during the past month [27]: (1) having no food in the household due to lack of resources; (2) a household member going to bed hungry due to insufficient food; and (3) a household member going a full day and night without eating. Each situation was scored according to its reported frequency: rarely (1-2 times, score = 1), sometimes (3-10 times, score = 2), or often (more than 10 times, score = 3). Scores were summed, and households were classified into three categories: mild hunger (total score ≤1), moderate hunger (total score 2-3), and severe hunger (total score 4-6).

The household meal distributor was defined as the individual responsible for deciding how prepared food was allocated among household members. This role could be fulfilled by the father, mother, or another household member (e.g., a sibling or domestic worker). Finally, daily meal frequency was measured by recording the number of meals consumed within the household per day. Meals eaten outside the home or separately by individual members were not included in this count.

**Data analysis:** after data collection, a cleaning process was implemented to detect outliers, correct data entry errors, and address missing values. The final dataset retained only validated entries, ensuring reliability for analysis.

Poisson regression models, chosen because the outcome variable represented discrete count data, were fitted separately for men and women to identify predictors of individual dietary diversity scores. The assumptions of the Poisson model, independence of observations, equidispersion, and absence of multicollinearity, were assessed prior to model estimation. To examine determinants of the gender difference in dietary diversity (DDS-WM), a multivariable linear regression model was applied. Linearity, homoscedasticity, normality of residuals, and multicollinearity were evaluated to ensure model adequacy. Due to the study´s cross-sectional design and low levels of missing data, no subgroup or sensitivity analyses were performed. A significance level of α ≤0.05 was used to determine statistical significance.

**Ethical considerations:** this study was approved by the Ethics Committee of the Kinshasa School of Public Health (Approval No.: ESP/CE/46/2024) as well as by provincial and local administrative and health authorities. Ethical principles were strictly followed throughout the study, with informed consent obtained from all participants. Confidentiality of all collected information was maintained at all times.

## Results

A total of 360 households were included in the analysis. Households in which the head was absent at the time of the initial investigator visit were subsequently revisited. In cases where the head declined participation, the household was replaced to ensure that the targeted sample size was maintained. There were no missing data for key variables related to household composition, dietary diversity, or socio-demographic characteristics. The majority of respondents were female (96%), and women also represented 59% of household heads. Most respondents were under 35 years of age (64%), with a mean age of 32 years (SD ±12), while household heads were predominantly aged 35 or older (71%), with a mean age of 43 years (SD ±13). Regarding education, 59% of respondents and 61% of household heads had completed secondary education. Trading was the most common occupation for both groups, reported by 38% of respondents and 47% of household heads. The average household size was six members. Just over half of the households (51%) were tenants on their residential plots. The distribution across wealth quintiles was approximately even, with a slight overrepresentation (21%) in the lowest quintile (Table 1).

**Table 1:** demographic characteristics of study participants, recruited from households in the Bonzola Health Zone (n=360)

Features	Respondents		Heads of household		Households	
	n	% (Cl95)	n	% (Cl95)	n	% (Cl95)
**Sex**						
Female	347	96.4 (93.9 - 98.1)	212	58.9 (53.6 - 64.0)	N / A	
Male	13	3.6 (1.9 - 6.1)	148	41.1 (36.0 - 46.4)	N / A	
Age groups						
<35	230	63.9 (58.7 - 68.9)	106	29.4 (24.8 - 34.4)	N / A	
≥35	130	36.1 (31.1 - 41.3)	254	70.6 (65.6 - 75.2)	N / A	
Age in completed years (mean ± standard deviation)	32.6±12.4		42.9±13.3		N / A	
**Respondent's level of education**						
No level of education	30	8.3 (5.7 - 11.7)	15	4.2 (2.4 - 6.8)	N / A	
Primary	72	20 (16.0 - 24.5)	64	17.8 (14.0 - 22.1)	N / A	
Secondary	214	59.4 (54.2 - 64.6)	219	60.8 (55.6 - 65.9)	N / A	
Superior	44	12.2 (9.0 - 16.1)	62	17.2 (13.5 - 21.5)	N / A	
**Respondent's profession**						
Shopkeeper	136	37.8 (32.7 - 43.0)	170	47.2 (42.0 - 52.5)	N / A	
Unemployed/ housewife	150	41.7 (36.5 - 46.9)	73	20.3 (16.2 - 24.8)	N / A	
Resourceful	41	11.4 (8.3 - 15.1)	41	11.4 (8.3 - 15.1)	N / A	
State civil servant	28	7.8 (5.2 - 11.0)	58	16.1 (12.5 - 20.3)	N / A	
MIBA agent	4	1.1 (0.3 - 2.8)	11	3.1 (1.5 - 5.4)	N / A	
Digger	1	0.3 (0.0 - 1.5)	7	1.9 (0.8 - 4.0)	N / A	
Household size (mean ± standard deviation)	N / A		N / A		6.2±2.2	
**Title/quality of occupation of the plot**						
Tenant	N / A		N / A		182	50.6 (45.3 - 55.8)
Owner	N / A		N / A		157	43.6 (38.4 - 48.9)
Family	N / A		N / A		18	5 (3.0 - 7.8)
Sub-housed	N / A		N / A		3	0.8 (0.2 - 2.4)
**Quintiles of the wealth index**						
Very low	N / A		N / A		76	21.1 (17.0 - 25.7)
Low	N / A		N / A		68	18.9 (15.0 - 23.3)
Middle	N / A		N / A		70	19.4 (15.5 - 23.9)
High	N / A		N / A		74	20.6 (16.5 - 25.1)
Very high	N / A		N / A		72	20 (16.0 - 24.5)

N / A: not applicable; MIBA: Bakwanga Mining Company

**Figure 1:**
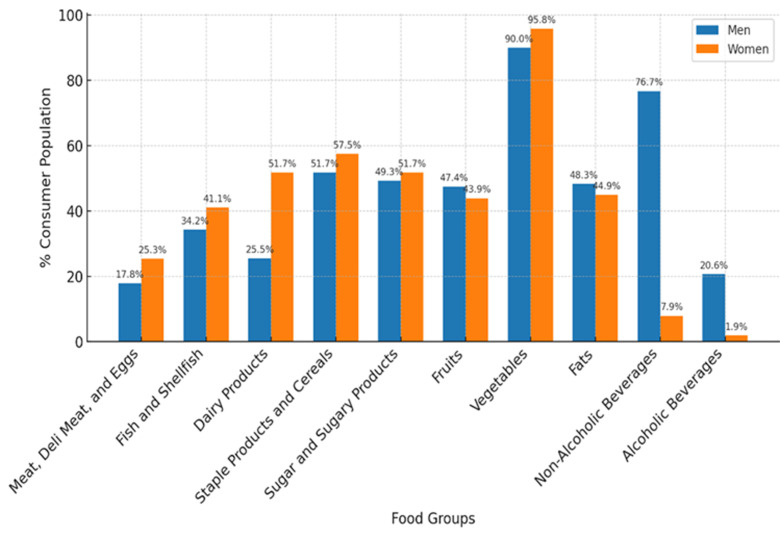
food groups diversity between men and women recruited from households in the Bonzola Health Zone (n=360)

Women generally consumed a greater variety of food groups over the seven days preceding the survey than men. The proportion of households reporting women's consumption of dairy products (51.7% vs 25.5%), fish and shellfish (41.1% vs 34.2%), meat, deli meat, and eggs (25.3% vs 17.8%) and vegetables (95.8% vs 90.0%) was higher than that for men. Conversely, men reported higher consumption of alcoholic beverages (20.6% vs 1.7%) and non-alcoholic drinks (76.7% vs 7.9%) (Figure 1). The Mann-Whitney U test demonstrated a statistically significant difference in dietary diversity between men and women. U= 59004.0; Z= -2.172, p= 0.030 (<0.05) (Table 2).

**Table 2:** dietary diversity scores (DDS) among men and women recruited from households in the Bonzola Health Zone (n=360)

Variables	DDS men	DDS women
Average rank	344.40	376.60
Sum of ranks	123984.0	135576.0
Mann-Whitney U-test	59004.0	
Z	-2.172	
p-value	0.030	

DDS: dietary diversity score

The Poisson regression model (generalized linear model) examining factors associated with men´s dietary diversity shows the men´s DDS increased by approximately 3% for each additional household member (β = 0.028, p < 0.05), but decreased by 8% with each one-unit increase in the household hunger index (β = -0.083, p < 0.05). Similarly, for women, dietary diversity was negatively associated with household hunger, showing a 9% reduction in DDS per unit increase in the hunger index (β = -0.091, p < 0.05) (Table 3).

**Table 3:** factors associated with dietary diversity based on the generalized linear model among men and women recruited from households in the Bonzola Health Zone (n=360)

Suspected factors	DDS women			DDS men		
	β	Stand Error.	p	β	Stand Error.	p
Wealth index	0.027	0.0245	0.264	0.040	0.0253	0.116
Household size	0.006	0.0130	0.628	0.028	0.0132	0.037
Title of occupation of the plot	0.024	0.0419	0.561	0.016	0.0437	0.711
Meal distributor	-0.002	0.0515	0.972	0.000	0.0523	0.996
Number of meals per day	-0.022	0.0546	0.685	0.011	0.0559	0.845
Hunger Index (HHS)	-0.091	0.0366	0.013	-0.083	0.0381	0.030
Coping Strategies Index (rCSI)	0.014	0.0087	0.100	0.010	0.0092	0.277
Occupation of the head of the household	-0.009	0.0200	0.658	0.009	0.0205	0.657
Level of education of the head of household	-0.029	0.0457	0.522	-0.00007	0.0476	0.999
Age of head of household	-0.001	0.0023	0.676	-0.003	0.0024	0.193
Gender of head of household	-0.030	0.0596	0.614	-0.095	0.0614	0.120

DDS: dietary diversity score

Generalized linear model analysis identified several factors associated with the difference in dietary diversity scores between women and men (DDS-WM) (adjusted R^2^= 0.047, p < 0.05). These included the wealth index, household size, number of meals per day, education level of the household head, and the reduced Coping Strategies Index (rCSI). The DDS-WM decreased with higher household wealth (β = -0.159, p < 0.05), larger household size (β = -0.139, p < 0.05), increased meal frequency (β = -0.127, p < 0.05), and higher education of the household head (β = -0.146, p < 0.05). In contrast, DDS-WM increased with higher rCSI scores (β = 0.100, p < 0.05). Multicollinearity was ruled out, as all tolerance values exceeded 0.1 and all variance inflation factors (VIFs) were below 10 (Table 4).

**Table 4:** factors associated with the difference in dietary diversity between women and men recruited from households in the Bonzola Health Zone (n=360)

Suspected factors	Difference between DDS women and DDS men			
	Pearson's β (Beta)	p-value	Adjusted R-squared	F (p-value)
Wealth index	-0.159	0.001	0.047	2.595 (0.003)
Household size	-0.139	0.004		
Title of the occupation of the plot	0.065	0.108		
Meal distributor in the household	-0.054	0.154		
Number of meals per day in the household	-0.127	0.008		
Household Hunger Index (HHS)	0.068	0.100		
Coping Strategies Index (rCSI)	0.100	0.029		
Occupation of the head of the household	0.016	0.379		
Level of education of the head of household	-0.146	0.003		
Age of head of household	0.045	0.196		
Gender of head of household	0.084	0.055		

DDS: dietary diversity score

## Discussion

This study aimed to assess the difference in dietary diversity between men and women in households in the Bonzola Health Zone and to identify factors associated with this difference. The findings revealed that women had significantly higher dietary diversity scores than men. A generalized linear model identified several factors associated with the gender gap in dietary diversity, including wealth index, household size, number of meals per day, education level of the household head, and the reduced Coping Strategies Index (rCSI). No collinearity was observed among these predictors, as confirmed by acceptable tolerance values and variance inflation factors.

With respect to eating habits, women were found to consume a broader range of food groups, including dairy products, vegetables, fish and shellfish, and animal-based proteins, in comparison to men. These results contrast with findings from patriarchal societies, where men, often as financial decision-makers, exhibit higher dietary diversity [19,28]. In Bonzola, however, the majority (59%) of household heads were women (compared to 41% men), which may explain the more equitable or even female-biased food distribution. This finding aligns with literature suggesting that in urban areas of the DRC, women often experience lower food insecurity due to greater autonomy in household food-related decisions [7]. Nonetheless, other studies have found no consistent association between the sex of the household head and dietary quality, underscoring the need for context-specific interpretations [17].

The household hunger index showed a significant inverse relationship with dietary diversity for both sexes. As household hunger increased, dietary diversity decreased by 9% in women and 8% in men. This result reinforces existing evidence that food insecurity severely constrains dietary quality, particularly for vulnerable household members [17,19,29]. Previous research has shown that in food-insecure settings, women are often the last to eat or receive smaller food portions, with adverse consequences for their health and nutrition [14,17].

Interestingly, men´s dietary diversity increased with household size, rising by approximately 3% for each additional household member. While this may appear counterintuitive, some studies suggest that larger households may benefit from pooled labor and income, enhancing food access [7]. However, this dynamic is context-dependent, as larger households may also experience diminished dietary diversity if economic resources do not scale with household growth [7]. It is also important to note that while the DDS-WM serves as a useful indicator of intra-household dietary differences, it has several limitations. As a difference-based measure, it reflects relative disparities between women and men but does not indicate whether overall dietary diversity is adequate. For example, households with uniformly low dietary diversity and those with uniformly high dietary diversity may show similar DDS-WM values. Furthermore, the indicator does not account for differences in energy requirements or portion sizes between individuals; it considers only the number of food groups consumed. Therefore, DDS-WM should be interpreted as a relative measure of dietary distribution within the household rather than a comprehensive assessment of nutritional equity.

The observed difference in dietary diversity between women and men was influenced by wealth, education, and meal frequency. A higher wealth index, a greater number of meals per day, and more educated household heads were all associated with a narrower gender gap in dietary diversity. These findings are consistent with prior studies showing that economic resources and education promote more diverse diets, especially for women who often manage food purchasing and preparation [30,31]. Conversely, the coping strategies index was positively associated with the gender gap. As food insecurity intensifies and households’ resort to extreme coping strategies, such as reducing portion sizes or skipping meals, women may adapt their own food intake patterns differently from men, potentially exacerbating disparities.

Overall, the finding that women had higher dietary diversity scores than men differs from much of the literature on gendered food allocation, which often reports disadvantages for women in intra-household food distribution. However, this result should be interpreted cautiously and within the specific context of the study population. Empirically, our data show that women consumed a greater variety of food groups than men. This pattern may reflect context-specific household dynamics rather than a broader reversal of established gender norms. For example, in this setting, women are typically responsible for food preparation and may have more direct access to diverse food items during cooking and meal planning. While this explanation is consistent with qualitative observations from similar contexts, our study did not directly measure intra-household allocation practices, and therefore, this interpretation remains a hypothesis rather than a confirmed mechanism.

Additionally, dietary diversity scores capture the number of food groups consumed but do not reflect portion sizes or caloric intake. It is therefore possible that men consumed larger quantities of fewer food groups, while women consumed smaller portions across a wider variety of foods. Without quantitative intake data, this possibility would not be confirmed.

Importantly, our findings should not be interpreted as evidence that gender-based nutritional inequities are absent in this context. Rather, they suggest that patterns of dietary diversity may differ from commonly reported allocation disadvantages, highlighting the need for context-specific investigation. Further research incorporating qualitative methods and detailed dietary intake assessments would help clarify the mechanisms underlying these observed differences.

**Strengths and limitations:** this study has several strengths. It used a representative sample of 360 households, contributing new evidence on gendered patterns in dietary diversity in an urban area of the DRC. The focus on intra-household differences adds important nuance to food security research, which often aggregates data at the household level. The use of robust statistical methods, including non-parametric testing and multivariable regression, supports the validity of the findings. Moreover, the study contributes to the evidence base for designing gender-sensitive nutrition interventions in fragile settings.

Nevertheless, some limitations should be acknowledged. First, the cross-sectional design prevents causal inference; the associations identified cannot establish temporal relationships. Second, the study relied on self-reported food recall rather than objective measures such as food weighing or nutrient analysis, which may introduce recall or reporting bias. Third, while the women´s dietary diversity score is validated and widely used, the equivalent for men has not been as rigorously tested, which may limit the reliability of gender comparisons. A further limitation relates to the predominance of female respondents in the study. As dietary data were self-reported, and women were more frequently responsible for food preparation and household interviews, this may have introduced differential reporting bias. Women may have provided more accurate information regarding their own dietary intake compared to that of men, potentially affecting the representativeness of male dietary diversity scores. This imbalance could have led to either overestimation or underestimation of the observed gender differences. Consequently, the findings regarding intra-household dietary disparities should be interpreted with caution. Future studies may benefit from ensuring direct individual-level dietary assessment for both men and women to reduce potential reporting bias.

Importantly, the study was conducted in a single urban health zone, which limits the generalizability of the findings to other settings within the DRC. Urban households may differ substantially from rural households in terms of market access, livelihood strategies, food availability, cultural norms, and gender roles related to food allocation. For example, urban residents often rely more heavily on purchased foods and cash income, whereas rural households may depend more on subsistence agriculture and seasonal production. These structural differences could influence both overall dietary diversity and intra-household food distribution patterns. Furthermore, levels of food insecurity, exposure to conflict, and socioeconomic heterogeneity vary considerably across provinces and between urban and rural areas in the DRC. As a result, the observed gender dynamics in dietary diversity may not reflect patterns in rural communities or in other urban contexts with different economic or cultural characteristics.

Lastly, the absence of qualitative data restricts deeper exploration of household food dynamics and individual preferences or power relations. Without qualitative insights, the mechanisms underlying the observed gender differences remain only partially understood. Future research integrating mixed-method approaches across diverse geographic settings, including rural and peri-urban areas, would strengthen the external validity of these findings and provide a more comprehensive understanding of context-specific gendered food allocation practices.

Despite these limitations, the study´s findings are relevant for nutrition policy and program design. The results highlight the importance of addressing intra-household disparities in dietary diversity and the potential value of targeting nutrition interventions at the household level with gender-sensitive components. The observed associations between dietary diversity and household-level socioeconomic factors also underscore the need for integrated approaches that combine food assistance with education, livelihoods, and resilience-building strategies.

## Conclusion

This study assessed intra-household differences in dietary diversity between men and women in the Bonzola Health Zone and identified associated determinants. Women had significantly higher dietary diversity scores than men. The gender gap was influenced by household wealth, education level of the household head, number of meals per day, household size, and the reduced Coping Strategies Index (rCSI). Higher household hunger was associated with lower dietary diversity for both sexes, underscoring the strong link between food insecurity and diet quality. No multicollinearity was observed among predictors, supporting the robustness of the model. These findings highlight the importance of gender-sensitive and context-specific nutrition interventions in food-insecure urban settings. Economic strengthening, improved educational attainment, and strategies to reduce reliance on negative coping mechanisms may contribute to more equitable dietary outcomes. Policies and programs addressing food insecurity in the Democratic Republic of the Congo should consider intra-household dynamics to ensure that improvements in food access translate into equitable nutritional benefits for both men and women.

### What is known about this topic

- Slum residency, large household size, poverty, low education levels, and older household heads continue to exacerbate food insecurity;
- Intra-household disparities, especially those based on gender, can significantly affect individual nutritional outcomes.

### What this study adds

- The significant differences in dietary diversity between men and women within households in the Bonzola Health Zone;
- The study highlights the critical role of factors such as wealth index, household size, and survival strategies in shaping dietary diversity outcomes.

## References

[1] Asma KM, Kotani K (2023). Intrahousehold Food Intake Inequality by Family Roles and Age Groups. Nutrients.

[2] Harris-Fry H, Shrestha N, Costello A, Saville NM (2017). Determinants of intra-household food allocation between adults in South Asia-a systematic review. Int J Equity Health.

[3] Food and Agriculture Organization (FAO) (2008). An Introduction to the Basic Concepts of Food Security.

[4] Food and Agriculture Organization (FAO) (2023). International Fund for Agricultural Development (IFAD), United Nations Children's Fund (UNICEF), World Food Program (WFP), World Health Organization (WHO). The State of Food Security and Nutrition in the World 2023: Urbanization, agrifood systems transformation and healthy diets across the rural-urban continuum.

[5] Njuki J, Sanginga PC (2013). Women, Livestock Ownership and Markets: Bridging the Gender Gap in Eastern and Southern Africa, Women, Livestock Ownership and Markets: Bridging the gender gap in Eastern and Southern Africa.

[6] IPC Democratic Republic of the Congo. Analyse IPC de l´insécurité alimentaire aiguë juillet, 2023 juin 2024 publié le 6 octobre.

[7] Institut National de la Statistique (INS) (2020). République Démocratique du Congo. Evaluation approfondie de la Sécurité Alimentaire en situation d´urgence (EFSA) dans les provinces de Kasaï Central et Kasai Oriental.

[8] Wang Z, Josephson A, Dizon F (2025). Intra-household inequality in food expenditures and diet quality in the Philippines. Food Security.

[9] Brown C, Van de Walle D (2021). Headship and poverty in Africa. The World Bank Economic Review.

[10] Hadley C, Lindstrom D, Tessema F, Belachew T (2008). Gender bias in the food insecurity experience of Ethiopian adolescents. Soc Sci Med.

[11] Harris-Fry HA, Paudel P, Shrestha N, Harrisson T, Beard BJ, Jha S (2018). Status and determinants of intra-household food allocation in rural Nepal. Eur J Clin Nutr.

[12] Li T, St-Germain AA, Tarasuk V (2023). Household Food Insecurity in Canada.

[13] Quisumbing AR, Maluccio J (1999). Intrahousehold allocation and gender relations: new empirical evidence (English).

[14] Ahmed A, Coleman F, Ghostlaw J, Hoddinott JF, Menon P, Parvin A (2022). Increasing production diversity and diet quality through agriculture, gender, and nutrition linkages: A cluster-randomized controlled trial in Bangladesh. Intl Food Policy Res Inst.

[15] Ministère de la Santé Publique Hygiène et Prévoyance Sociale République Démocratique du Congo. Estimations de mobilité et population : Kasaï Oriental.

[16] Chow SC, Shao J, Wang H, Lokhnygina Y (2017). Sample size calculations in clinical research.

[17] Alam MJ, Begum IA, Mastura T, Kishore A, Woodhill J, Chatterjee K (2023). Agricultural diversification and intra-household dietary diversity: Panel data analysis of farm households in Bangladesh. PLoS One.

[18] Waseem M, Li X, Jamil I, Islam AH, Abbas Q, Raza MH (2023). Do crop diversity and livestock production improve smallholder intra-household dietary diversity, nutrition and sustainable food production?. Empirical evidence from Pakistan. Frontiers in Sustainable Food Systems.

[19] Singh S, Jones AD, DeFries RS, Jain M (2020). The association between crop and income diversity and farmer intra-household dietary diversity in India: Singh S et al. Food Security.

[20] Dehghan M, Ilow R, Zatonska K, Szuba A, Zhang X, Mente A (2012). Development, reproducibility and validity of the food frequency questionnaire in the Poland arm of the Prospective Urban and Rural Epidemiological (PURE) study. J Hum Nutr Diet.

[21] Correa Guzmán N CBV, Sepúlveda Herrera DM, Cárdenas Sánchez DL, Manjarrés Correa LM (2022). Validation of an instrument to assess food diversity in women of childbearing age in Medellín, Colombia. Public Health Nutr.

[22] Food and Agriculture Organization (FAO) (2021). Minimum dietary diversity for women: An updated guide to measurement - from collection to action.

[23] Filmer D, Pritchett LH (2001). Estimating wealth effects without expenditure data--or tears: an application to educational enrollments in states of India. Demography.

[24] Rutstein SO, Johnson K (2004). DHS Comparative Report 6: The DHS wealth index.

[25] Maxwell D, Caldwell R (2008). Coping Strategies Index: Field Methods Manual.

[26] Deitchler M, Ballard T, Swindale A, Coates J (2010). Validation of a measure of household hunger for cross-cultural use.

[27] World Food Programme (WFP) (2014). Indicateurs de la sécurité alimentaire.

[28] Aurino E, Fernandes M, Penny ME (2017). The nutrition transition and adolescents' diets in low-and middle-income countries: a cross-cohort comparison. Public Health Nutr.

[29] Akerele D (2011). Intra-household food distribution patterns and calorie inadequacy in South-Western Nigeria. International journal of consumer studies.

[30] Lepiller O, Fournier T, Bricas N, Figuié M (2021). Méthodes d'investigation de l'alimentation et des mangeurs.

[31] Marla KS, Padmaja R (2023). Analyzing gender differentials in dietary diversity across urban and peri-urban areas of Hyderabad, India. BMC Nutr.

